# Splicing Mutations Impairing CDKL5 Expression and Activity Can be Efficiently Rescued by U1snRNA-Based Therapy

**DOI:** 10.3390/ijms20174130

**Published:** 2019-08-24

**Authors:** Dario Balestra, Domenico Giorgio, Matteo Bizzotto, Maria Fazzari, Bruria Ben Zeev, Mirko Pinotti, Nicoletta Landsberger, Angelisa Frasca

**Affiliations:** 1 Department of Life Sciences and Biotechnology, University of Ferrara, 44121 Ferrara, Italy; 2 Department of Medical Biotechnology and Translational Medicine, University of Milan, 20090 Milan, Italy; 3 Pediatric Neurology Unit, Edmond and Lily Safra Pediatric Hospital, Sheba Medical Center and Sackler School of Medicine, Tel Aviv University, 61000 Tel Aviv, Israel

**Keywords:** splicing, U1snRNA, CDKL5, mutations

## Abstract

Mutations in the *CDKL5* gene lead to an incurable rare neurological condition characterized by the onset of seizures in the first weeks of life and severe intellectual disability. Replacement gene or protein therapies could represent intriguing options, however, their application may be inhibited by the recent demonstration that *CDKL5* is dosage sensitive. Conversely, correction approaches acting on pre-mRNA splicing would preserve *CDKL5* physiological regulation. Since ~15% of *CDKL5* pathogenic mutations are candidates to affect splicing, we evaluated the capability of variants of the spliceosomal U1 small nuclear RNA (U1snRNA) to correct mutations affecting +1 and +5 nucleotides at the 5′ donor splice site and predicted to cause exon skipping. Our results show that *CDKL5* minigene variants expressed in mammalian cells are a valid approach to assess *CDKL5* splicing pattern. The expression of engineered U1snRNA effectively rescued mutations at +5 but not at the +1 nucleotides. Importantly, we proved that U1snRNA-mediated splicing correction fully restores CDKL5 protein synthesis, subcellular distribution and kinase activity. Eventually, by correcting aberrant splicing of an exogenously expressed splicing-competent *CDKL5* transgene, we provided insights on the morphological rescue of *CDKL5* null neurons, reporting the first proof-of-concept of the therapeutic value of U1snRNA-mediated *CDKL5* splicing correction.

## 1. Introduction

Mutations in the X-linked cyclin dependent kinase like gene (*CDKL5*; *OMIM 300672*, *300203*) cause a broad spectrum of neuropsychiatric disorders that affect both genders and generally share the common features of severe intellectual disability and early drug-resistant epilepsy, emerging in the first months of life [1]. No cure is available to combat the primary pathology for *CDKL5*-deficiency disorder (CDD) and treatment is based on supportive therapy for the comorbidities, and on rehabilitation. Replacement gene and protein therapy could be an intriguing option, but this approach might be hampered by the need to preserve the finely regulated control of *CDKL5* levels since *CDKL5* is dosage sensitive, as suggested by the existence of a *CDKL5* duplication syndrome [2].

In this peculiar scenario, correction approaches acting on pre-mRNA splicing would be of great interest for CDD since they would preserve the natural gene regulation and, even with the strongest correction effect, would not boost the expression above the physiological levels. It is worth noting that a relevant proportion (~15%) of gene variants associated with CDD are candidates to affect splicing (http://mecp2.chw.edu.au/cdkl5/cdkl5_variant_list_copy.php) and this fraction is largely underestimated, considering that missense changes can impair this process [3,4,5,6].

In the last decade, we and others have demonstrated that exon-skipping, certainly the most frequent aberrant splicing mutations’ outcome, can be efficiently counteracted by variants of the U1 small nuclear RNA (U1snRNA), the RNA component of the key spliceosomal U1 ribonucleoprotein (U1snRNP) that mediates the 5′ splice site (5′ss) recognition and exon definition in the earliest splicing step [7]. In addition, we have discovered a second-generation of U1snRNA variants that elicit their effect targeting intronic regions downstream of the 5′ss (Exon Specific U1snRNA, ExSpeU1), thus increasing gene specificity [8]. Remarkably, by using cellular and mouse models of human diseases, we have shown that a unique ExSpeU1 can rescue multiple exon-skipping mutations, either at the 5′ss, 3′ss or within the exon, thus expanding patient applicability [3,4,9,10,11,12,13,14,15,16,17].

Here, we examined the molecular mechanisms underlying aberrant *CDKL5* splicing and demonstrated their association with *CDKL5* deficiency. Most importantly, we developed U1snRNA variants able to rescue *CDKL5* mRNA splicing, protein synthesis and function. Accordingly, the morphology of *Cdkl5* null primary neurons is significantly ameliorated only when the expression of a *CDKL5* splicing defective cDNA is combined with the presence of the therapeutic U1snRNA. To our knowledge, this is the first proof-of-concept of a splicing correction strategy applied to *CDKL5*.

## 2. Results

**Figure 1:**
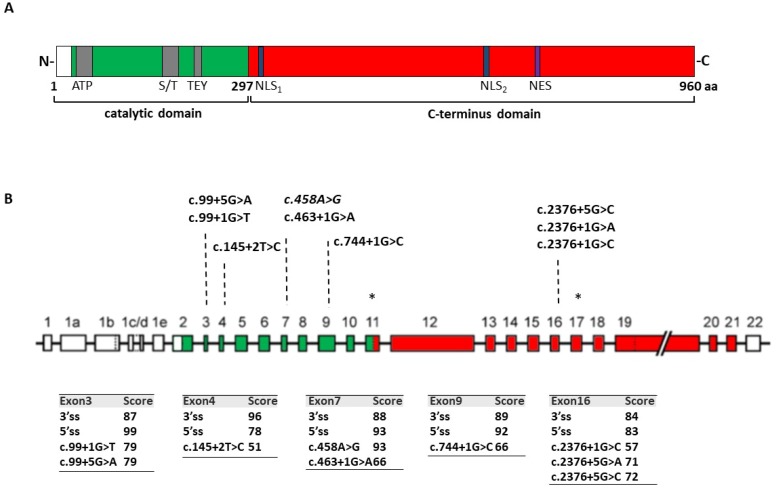
Schematic representation of the *CDKL5* gene and protein. (**A**) Schematic representation of the human isoform hCDKL5-1 [18] with its principal functional domains. The catalytic domain is shown in green while the included ATP binding site (ATP), the Ser/Thr kinase active site (S/T) and the Thr-Glu-Tyr (TEY) motif are highlighted in grey. The C-terminus domain is depicted in red and the nuclear localization signals (NLS_1_ and NLS_2_) and nuclear export signal (NES) are in blue and violet, respectively. (**B**) Schematic representation of the *CDKL5* gene with introns and exons represented respectively by lines and boxes. Nucleotide variants investigated in this study are reported in the upper part of the figure. Asterisks denote the exons whose presence differs between different transcript isoforms. The bioinformatics prediction of 3′ and 5′ss scores for each exon in normal and pathological condition is reported in the lower part of the panel.

Through computational analysis (www.umd.be/HSF/) of the nucleotide changes annotated in the *CDKL5* variation database (http://mecp2.chw.edu.au/cdkl5/cdkl5_variant_list_copy.php), we selected variants expected to affect splicing. Among them, as representative models, we focused on those changes that are predicted to weaken the authentic 5′ss of exons 3, 4, 7, and 9, which encode the key catalytic domain of *CDKL5*. Furthermore, since several disease-associated changes occur at the 5′ss of exon 16, this exon was also investigated (Figure 1A,B).

Due to the impossibility to directly evaluate *CDKL5* splicing in the brain that represents the tissue mainly involved in the disorder [18], the effects of the selected mutations were analyzed through minigene expression studies, representing a well-established technique to investigate splicing mechanisms and, for one mutation, also in immortalized lymphocytes.

### 2.1. Ex Vivo Studies Indicate that the c.2376+5G>A Change Induces Exon 16 Skipping and Nonsense-Mediated mRNA Decay

**Figure 2:**
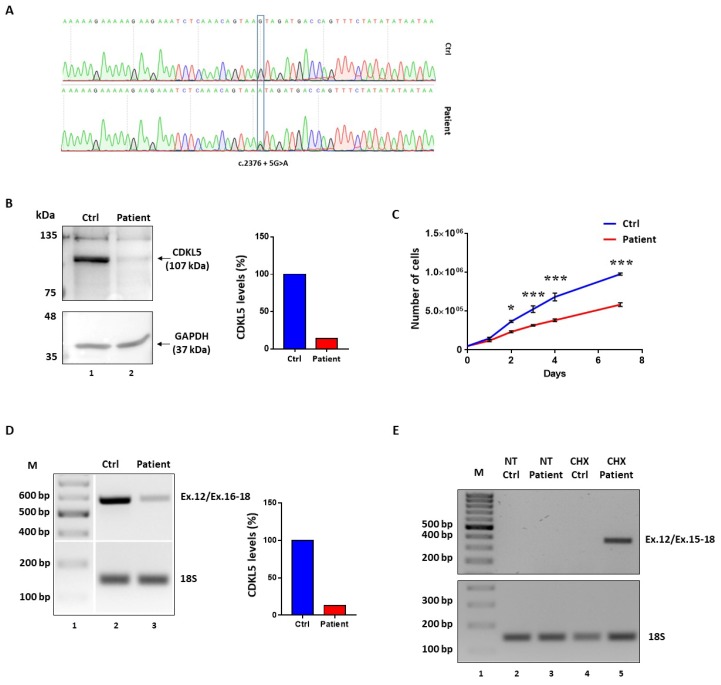
Ex vivo studies indicate that the c.2376+5G>A variant is associated with exon 16 skipping and nonsense-mediated mRNA decay. (**A**) Electropherograms showing the sequence of *CDKL5* exon 16 in a control (Ctrl) or a heterozygous patient harboring the c.2376+5G>A mutation obtained from the genomic DNA of the corresponding lymphoblastoid cell lines. The position of the mutation is indicated by a blue rectangle. (**B**) Western blotting analysis of CDKL5 expression in the immortalized lymphocytes from the control (lane 1) or the *CDKL5*-deficiency disorder (CDD) patient (lane 2). Histograms report the amount of CDKL5 protein isoform calculated by densitometric analysis of bands and normalized to GAPDH. (**C**) Growth rate curve of immortalized lymphocytes from the control donor or the CDKL5 patient. Asterisks indicate a significant difference between the number of control and patient’s cells at the selected time points. * *p* < 0.05; *** *p* < 0.001 by two-way repeated measure ANOVA, followed by Sidak’s multiple comparison test. (**D**) Reverse transcriptase (RT)-PCR evaluating the splicing pattern of *CDKL5* exon 16 in the immortalized lymphocytes using the forward primer located in exon 12 and the reverse primer binding the exon 16–18 junction. Levels of 18S rRNA were used as internal standard. Histograms report the relative amount of correctly spliced *CDKL5* mRNA in patient’s cells with respect to control, arbitrarily set at 100%. (**E**) Evaluation of *CDKL5* exon 16 skipping transcripts after 4 h of cycloheximide treatment (CHX; 300 mg/mL) by RT-PCR using the forward primer located in exon 12 and the reverse primer binding the exon 15–18 junction. Thus, 18S amplification was used as internal standard.

Immortalized lymphocytes from a patient heterozygous for the c.2376+5G>A change (Figure 2A) were available for the study. From birth the patient suffers from severe epileptic disorder, characterized by various seizure types, which are resistant to medications and she has a profound intellectual disability [19].

Western blotting analysis in lymphocyte lysates revealed a remarkable reduction (~80%) of CDKL5 protein levels as compared to wild-type lymphocytes (Figure 2B). This was associated with a lower growth rate compared to control cells, with discrepancy detectable from day 2 to day 7 (* *p* < 0.05; *** *p* < 0.001; by two-way repeated measures ANOVA, followed by Sidak multiple comparison test; Figure 2C).

The evaluation of the *CDKL5* splicing patterns by reverse transcriptase (RT)-PCR with oligonucleotides spanning exons 12–18 did not reveal aberrantly spliced transcripts in *CDKL5* deficient lymphocytes but a remarkable reduction (~80%) of the correctly spliced transcripts (Figure 2D). To verify the occurrence of nonsense-mediated mRNA decay (NMD), which might prevent to appreciate aberrant and out-of-frame transcripts such as those arising from the most probable event of exon 16 skipping, we evaluated the splicing patterns in lymphocytes treated with cycloheximide (CHX), a well-established NMD inhibitor. As shown in Figure 2E, the RT-PCR with primers designed to exclusively detect exon 16 skipped transcripts provided an appreciable band only in the patient’s lymphocytes upon CHX treatment, but not in control cells.

Taken together, these data indicate that the c.2376+5G>A change is associated with skipping of exon 16, which leads to frameshift and insertion of a premature stop codon in exon 18, ultimately leading to NMD. This causes a remarkable reduction of *CDKL5* expression, which explains the associated CDD.

### 2.2. Minigene Expression Assays Demonstrate that Nucleotide Changes at 5′ss Mainly Induce Exon Skipping

**Figure 3:**
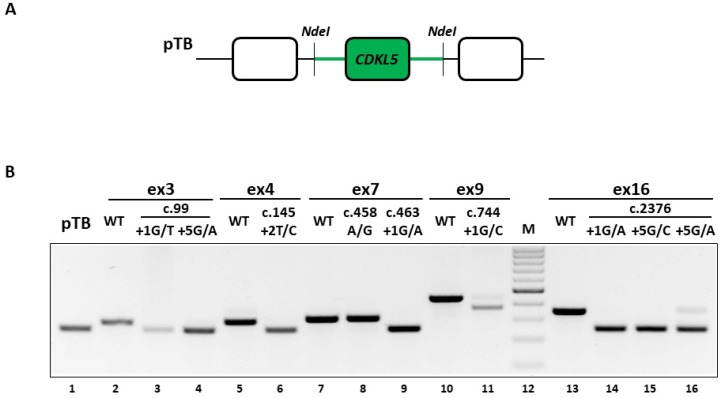
Splicing pattern analysis of *CDKL5* variants. (**A**) Schematic representation of a *CDKL5* minigene cloned into the pTB vector. Exonic and intronic sequences are represented by boxes and lines, respectively. Sequences of the pTB vector are depicted with empty boxes and black lines, while the *CDKL5* minigene is shown in green. As indicated, the minigene was cloned within the NdeI restriction site of the pTB plasmid. (**B**) RT-PCR analysis of the *CDKL5* minigene (wild-type or mutated) splicing pattern in HEK293T cells. Amplified products were separated on 2% agarose gel.

To unequivocally demonstrate the causative role of the selected nucleotide changes, we expressed the *CDKL5* minigene variants in mammalian cells and assessed their splicing patterns. Each *CDKL5* minigene construct contains the exon of interest and the surrounding intronic sequences (~500 bp) cloned in the exon-trapping pTB construct (Figure 3A). The expression of the wild-type minigenes in human embryonic kidney (HEK293T) cells indicated that each exon is properly spliced, as shown by the complete inclusion in the mature mRNA (Figure 3B, lanes 2, 5, 7, 10 and 13), which validated the experimental system.

Firstly, we investigated the impact of the c.2376+5G>A change, reporting the occurrence of exon 16 skipping (Figure 3B, lanes 16), a finding that is consistent with the ex vivo data. Moreover, the splicing assays demonstrated that the mutation is also compatible with trace levels of correctly spliced transcripts (8.2 ± 1.2% evaluated by densitometric analysis of bands), an information not obtainable in the patient’s lymphocytes, due to the confounding effect of the wild-type allele.

The good accordance of results between the ex vivo and in vitro data for the c.2376+5G>A variant prompted us to extend the splicing assays to the complete panel of selected mutations. As shown in Figure 3B and confirmed by DNA sequencing of amplicons, all changes but the c.458A>G and the c.744+1G>C variants led to exon-skipping. In particular the c.458A>G change (lane 8), which was expected to affect splicing because of its location into a putative exonic splicing enhancer (data not shown), did not alter the splicing process. On the other hand, the c.744+1G>C change (lane 11) appeared to be also compatible with traces of correctly spliced transcripts, which, however are attributable to the usage of an adjacent exonic cryptic 5′ss, thus resulting in an aberrant slightly longer form (+7 nucleotides). Moreover, the c.744+1G>C change was associated with a shorter transcript arising from the usage of a cryptic 5′ss located 70 nucleotides upstream of the authentic one, and predicted to encode a CDKL5 protein isoform lacking 23 amino acids.

Taken together, these data indicate the causative role of most mutations that trigger exon skipping and their association with *CDKL5* deficiency, and provide a reliable experimental system to explore correction approaches.

### 2.3. Engineered U1snRNAs Rescue Splicing Variants at +5 Positions in the Context of CDKL5 Exons 3 and 16

**Figure 4:**
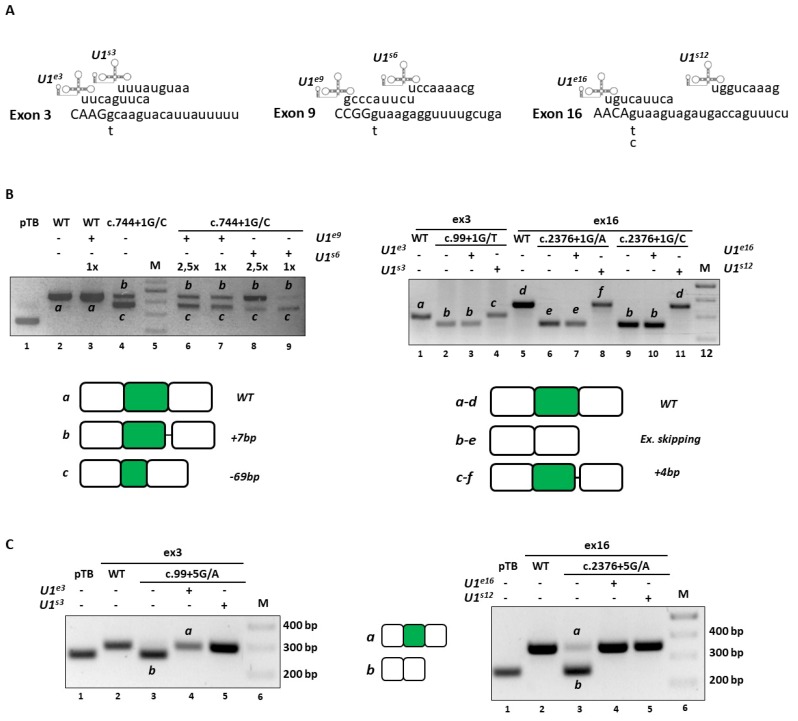
U1snRNA-mediated rescue of *CDKL5* splicing variants at position +1 and +5 of 5′ss. (**A**) Schematic representation of U1snRNA variants designed to restore proper *CDKL5* exon definition. Exonic and intronic sequences are reported in upper and lower cases, respectively. The sequence of 5′ tail of modified U1snRNA is reported on top. The presence of nucleotide changes, for each exon context, is reported below. U1^s3-s6-s12^ indicate the compensatory U1snRNA, whereas U1^e3-e6-e16^ refer to the ExSpeU1. (**B**) RT-PCR evaluation of the splicing patterns in HEK293T cells transiently transfected with *CDKL5* minigenes alone or in combination with a molar excess of engineered U1-expression plasmids. Each transcript is indicated by a letter and the corresponding schematic representation (with exons not in scale), validated by sequencing and depicting the WT and the resulting transcripts, is reported below. (**C**) Testing by RT-PCR, the *CDKL5* splicing patterns in HEK293T cells transfected with mutant minigenes alone or in combination with a molar excess (1.5×) of engineered U1-expression plasmids. Amplified products were separated on 2% agarose gel. The schematic representation of all resulting transcripts is reported in the middle.

Based on our previous studies [3,6,7,8,10,11,16,20], we expressed U1snRNA variants with the 5′tail designed to be perfectly complementary to the authentic 5′ss (compensatory U1snRNA) of each *CDKL5* exon or to downstream and less conserved intronic sequences (ExSpeU1) (Figure 4A).

While the expression of the c.744+1G>C mutant together with the compensatory U1snRNA favored the usage of an upstream exonic cryptic 5′ss, the ExSpeU1 induced the usage of a cryptic 5′ss located 8 nucleotides downstream the mutated 5′ss (Figure 4B), with partial intron retention that would lead to frameshift and insertion of a premature stop codon at position c.763.

Concerning the c.99+1G>T, c.2376+1G>A and c.2376+1G>C variants, the co-expression of the corresponding compensatory U1snRNA was ineffective, whereas the ExSpeU1 variants led to the usage of cryptic 5′ss located 5 nucleotides downstream of the mutated 5′ss (Figure 4B). The consequent partial intron retentions would lead to frame shifting and insertion of a premature stop codon respectively at position c.112 and c.2401.

Analogously, we challenged the c.99+5G>A and c.2376+5G>A mutants. The splicing analysis in cells co-expressing the *CDKL5* minigene variants with the compensatory U1snRNA or the ExSpeU1 revealed a complete splicing rescue as indicated by the exclusive presence of correct transcripts including exon 3 or 16 (Figure 4C). Notably, we confirmed these results in differentiated neuronal SHSY cell lines, that better represent the cell type mainly affected by the *CDKL5* deficiency (data not shown).

Taken together these data show that U1snRNA variants are effective on mutations at +5 positions but not on variants at the conserved +1 nucleotide.

### 2.4. The U1snRNA-mediated Splicing Correction Results in Restoration of CDKL5 Protein Synthesis and Activity

**Figure 5:**
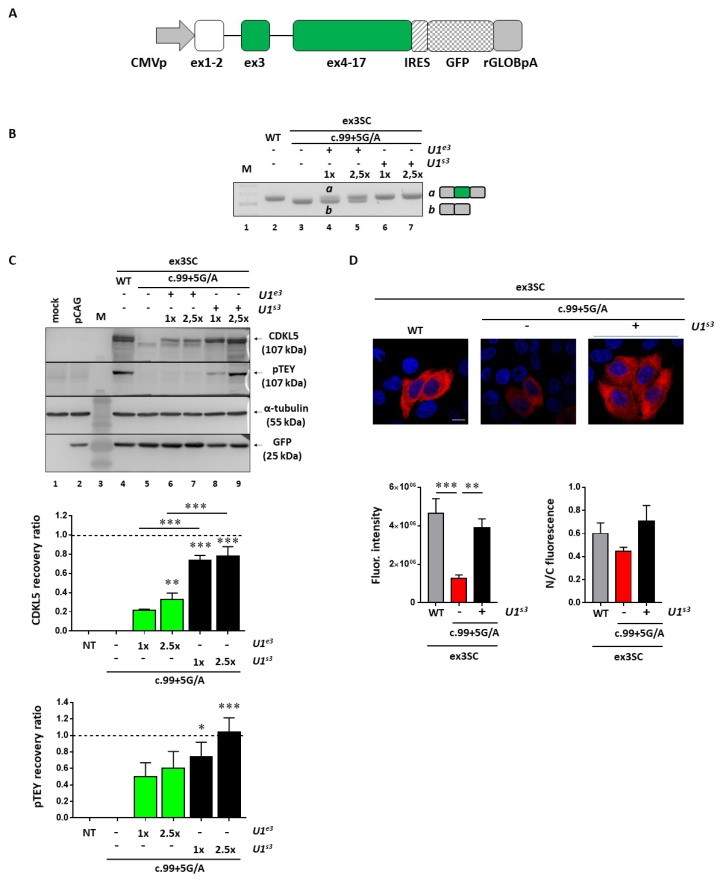
U1-mediated rescue of CDKL5 expression at the protein level. (**A**) Schematic representation of the full-length splicing-competent *CDKL5* expression cassette (CDKL5SC) used to evaluate the U1-mediated rescue of the c.99+5G>A mutation. (**B**) RT-PCR evaluation of the *CDKL5* splicing patterns in HEK293T cells transiently transfected with CDKL5SC alone or in combination with modified U1snRNAs (the compensatory U1snRNA (U1^s3^) and the ExSpeU1 (U1^e3^)). The schematic representation of the transcripts is reported on the right. (**C**) Western blotting analysis of U1-mediated rescue of CDKL5 expression and phosphorylation of its TEY motif in lysates of HEK293T cells transfected as in (**B**). The histograms show the mean ± SEM of the expression levels of full-length CDKL5 (above) normalized to GFP and TEY phosphorylation (below), calculated as a ratio between phospho-TEY and total CDKL5, in cells transfected with modified U1snRNAs (U1^e3^ and U1^s3^) and compared to cells transfected with c.99+5G/A mutant cDNA. NT indicates not transfected cells. α-tubulin served as loading control. Data were analyzed by one-way ANOVA, followed by Tukey’s multiple comparison test. When not properly indicated, asterisks denote a significant difference compared to cells transfected with c.99+5G/A mutant cDNA. * *p* < 0.05; ** *p* < 0.01; *** *p* < 0.001. (**D**) Immunofluorescence analysis of CDKL5 (in red) reporting its subcellular distribution in HeLa cells transfected with the WT or splicing defective (c.99+5G/A) CDKL5SC vector alone or in combination with the ExSpeU1snRNA (U1^s3^). Nuclei are identified by 4′,6-diamidino-2-phenylindole (DAPI) staining (in blue). Scale bar 10 µm. Histograms depict the fluorescence intensity and the nuclear/cytosolic distribution of WT, mutant CDKL5 alone or in combination with U1^s3^. Data are indicated as the mean ± SEM of the percentages of the calculated integrated density with respect to WT. ** *p* < 0.01; *** *p* < 0.001 by one-way ANOVA, followed by Tukey’s multiple comparison test.

To evaluate the impact of the splicing rescue on CDKL5 activity, we selected the c.99+5G>A mutation affecting the N-terminal catalytic domain as an informative model; we exploited a full-length splicing-competent *CDKL5* minigene in which exon 3 and the surrounding introns are placed into the h*CDKL5_1* full-length cDNA (Figure 5A). As expected, in transient transfection experiments in HEK293T cells, this construct drove the synthesis of correctly spliced *CDKL5* transcripts (Figure 5B, lane 2) that were paralleled by the synthesis of CDKL5 protein (Figure 5C, lane 4). Moreover, pTEY assays, conventionally used to assess the CDKL5 kinase activity [21], demonstrated that the recombinant CDKL5 protein possessed an appreciable catalytic activity. Conversely, the c.99+5G>A mutation led to complete exon 3 skipping (Figure 5B, lane 3) and to a shorter protein which is completely dysfunctional (Figure 5C, lane 5). Co-expression of the pSC-CDKL599+5A construct with the engineered U1snRNA resulted in the appreciable synthesis of normally spliced transcripts (Figure 5B, lanes 4-7). While the compensatory U1snRNA ameliorated *CDKL5* splicing in a dose-dependent manner, the ExSpeU1snRNA fully restored proper exon definition at the lowest dose (Figure 5B, compare lane 4 with 6). The rescue of splicing was clearly paralleled by the synthesis of the correct and catalytically active CDKL5 (Figure 5C, lanes 6–9). In particular, we observed a significant increase of CDKL5 protein in a dose-dependent manner upon co-transfection of U1snRNA variants, with the ExSpeU1 associated with the higher rescue efficiency (>70%, ** *p* < 0.01; *** *p* < 0.001 by one-way ANOVA, followed by Tukey’s multiple comparison test). Strikingly, a significant and dose-dependent increase of CDKL5 activity was also detected (Figure 5C), as indicated by the pTEY assay (* *p* < 0.05; *** *p* < 0.001 by one-way ANOVA, followed by Tukey’s multiple comparison test).

To provide further evidence for rescue, and in particular, mediated by the most effective U1snRNA variant, we analyzed the CDKL5 subcellular distribution by immunofluorescence. In good accordance with the western blotting results, the analysis of the immunofluorescence intensity showed a significant decrease of CDKL5 protein in cells transfected with the pSC-CDKL599+5A vector compared with cells transfected with the wild-type vector (** *p* < 0.01; *** *p* < 0.001 by one-way ANOVA, followed by Tukey’s multiple comparison test) (Figure 5D). The ExSpeU1snRNA rescued the CDKL5 expression to a level comparable with the wild-type condition. On the other hand, we did not observe a significant difference in the CDKL5 subcellular distribution, measured as the nuclear/cytoplasmic fluorescence ratio.

Taken together, these data prove that the splicing rescue mediated by the ExSpeU1 is paralleled by the restoration of proper protein biosynthesis and activity.

### 2.5. Cdkl5 Null Primary Neurons Are Morphologically Rescued if ExSpeU1 Restores CDKL5 Splicing

**Figure 6:**
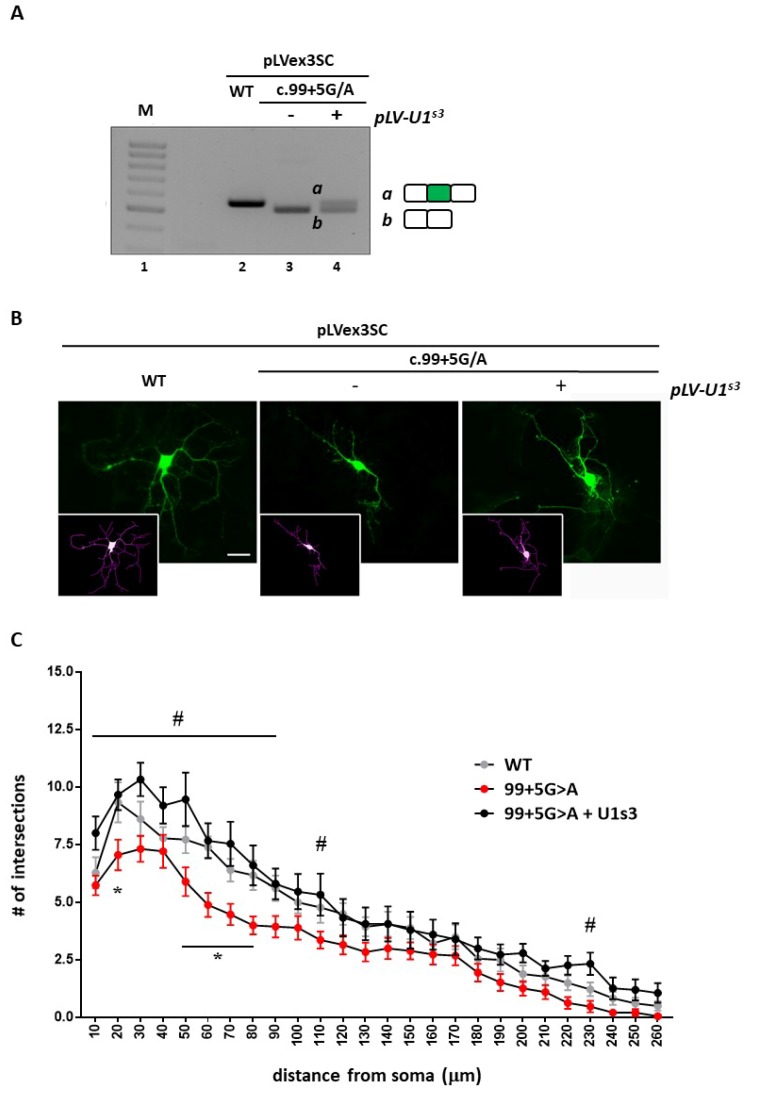
U1^s3^snRNA rescues the dendritic morphology of *Cdkl5* null hippocampal neurons. (**A**) RT-PCR analysis of *CDKL5* splicing in hippocampal WT neurons infected at DIV1 with lentiviruses expressing either the WT (pLLSC-CDKL5WT) or the mutant pLLSC-CDKL599+5A (c.99+5G>A) cDNA alone or in combination with lentiviruses expressing ExSpeU1^S3^ (pLV-U1^s3^). (**B**) Representative immunofluorescences of GFP positive *Cdkl5* null neurons (in green) and the corresponding masks (in violet) are reported. Neurons were infected at DIV1 with the WT or the mutant pLLSC-CDKL599+5A construct alone or in combination with viruses expressing ExSpeU1^S3^, and morphologically analyzed by Sholl analysis plug-in. Scale bar 20 µm. (**C**) The graph shows the mean ± SEM of the numbers of intersections of neurons of each experimental group. Data were analyzed by two-way ANOVA, followed by Tukey’s multiple comparison test. * *p* < 0.05 indicates a significant difference between c.99+5G>A and WT vector infected neurons; # *p* < 0.05 indicates a significant difference between c.99+5G>A and c.99+5G>A + pLV-U1^s3^.

Since CDD is a neurodevelopmental disease, we proceeded by analyzing whether correction of aberrant splicing of an exogenously expressed *CDKL5* could rescue typical phenotypes of mouse *Cdkl5* null neurons [22,23]. To this purpose, by infecting DIV1 wild-type hippocampal neurons with lentiviruses expressing either the wild-type splicing competent cDNA (pLLSC-CDKL5WT) or the c.99+5G>A mutant cDNA (pLLSC-CDKL599+5A), alone or together with lentiviruses expressing the ExSpeU1^S3^, we initially confirmed that the c.99+5G>A mutation induced exon skipping that can be rescued by the ExSpeU1 (Figure 6A).

We then proceeded to infect DIV1 *Cdkl5* null hippocampal neurons with lentiviruses, expressing the splicing-competent *CDKL5* variants and we analyzed their arborization at DIV5 (Figure 6B,C). *Cdkl5* null neurons infected with the pLLSC-CDKL599+5A virus showed a significant decrease in neuronal arborization with respect to neurons infected with the wild-type *CDKL5* construct. Although a trend toward reduction was observed for any distance from the soma, a statistically significant effect was mainly observed on shorter neurites (* *p* < 0.05 by two-way ANOVA followed by Tukey’s multiple comparison test). Strikingly, co-transduction of the virus expressing the ExSpeU1^S3^ increased the number of intersections at any distance from the soma therefore exhibiting a strong rescuing effect (^#^ *p* < 0.05 by two-way ANOVA followed by followed by Tukey’s multiple comparison test) (Figure 6C).

## 3. Discussion

At present, *CDKL5* deficiency is a severe genetic condition with no cure. Conventional augmentative gene therapy could represent an intriguing option, but its development might be complicated by the need of a finely regulated *CDKL5* expression, as suggested by the existence of a *CDKL5* duplication syndrome [2]. In this light, correction approaches acting at the post-transcriptional level would be of great interest for *CDKL5* deficiency since they would preserve the physiological gene regulation. Among them, induction of ribosome read-through by aminoglycoside drugs is an attractive option, as recently demonstrated for mutations introducing premature termination codons (PTCs) and reported in ~15% of patients with *CDKL5* deficiency [21]. However, the overall low efficiency of this strategy and the impaired CDKL5 catalytic activity of the rescued protein suggest caution for the translatability of the nonsense suppression therapy. On the other hand, intervention at pre-mRNA level through modulation of splicing by delivery of engineered U1snRNAs has been successfully exploited in several cellular and animal models of human disease [8,9,14,17,24], providing evidence that one single molecule can rescue multiple mutations, including nucleotide changes at 5′ss, 3′ss as well as within exons. It is worth mentioning that, by not affecting the transcriptional process, this approach would not boost *CDKL5* expression above the physiological levels, even in the presence of the strongest correction effect.

Here, through the minigene expression approach, we investigated the molecular mechanisms leading to *CDKL5* deficiency in the presence of the *CDKL5* c.99+1G>T, c.99+5G>A, c.145+2T>C, c.463+1G>A, c.744+1G>C, c.2376+1G>C, c.2375+5G>A and c.2376+5G>C nucleotide variants. Moreover, since missense mutations can also impair splicing [4], we included in the study the missense c.458A>G change, bio-informatically predicted to alter splicing. In particular, the splicing pattern analysis revealed that, whereas the c.458A>G change does not impact splicing, the c.744+1G>C, c.145+2T>C, c.463+1G>A, c.2376+1G>C and c.2376+5G>C changes are associated with aberrant transcripts (exon skipping or partial intron retention), that might account, if translated, for shorter and frame shifted CDKL5 protein isoforms. On the other hand, expression of minigene with the c.99+1G>T, c.99+5G>A and c.744+1G>C variants revealed the presence of aberrant transcripts (respectively exon skipping and partial exon deletion) that would code for an in-frame but shorter CDKL5 protein isoform. In all cases, the splicing pattern was in accordance with patients’ phenotypes and confirmed the pathogenic role of these variants in determining *CDKL5* deficiency. The molecular mechanisms underlying *CDKL5* deficiency were also investigated in immortalized lymphocytes of a heterozygous female patient, carrying the splicing defective c.2376+5G>A allele. While these studies confirmed the *CDKL5* splicing pattern observed in vitro, thus supporting the exploitation of the minigene approach for diagnostic purposes, they also highlighted the involvement of NMD, indicating the value of future studies aimed at investigating the therapeutic potential of inhibiting NMD for *CDKL5* mutations affecting its terminal portions. 

The knowledge of the aberrant splicing mechanisms led us to explore the U1snRNA-mediated approach, a strategy that could maintain the fine regulated control of *CDKL5* expression. Here, the delivery of both the compensatory U1snRNA or the second generation of U1snRNA variants, named Exon Specific U1snRNA (ExSpeU1), failed to restore exon definition in the presence of nucleotide changes occurring at position +1, in accordance with its crucial role within the 5′ss [25]. On the other hand, both U1snRNA variants completely rescued exon definition in the presence of c.99+5G>A and c.2376+5G>A variants, respectively located in the exon 3 and 16 contexts. It is worth noting the ability of U1snRNA variants to rescue nucleotide changes at position +5, but their failure for changes at position +1 of the same 5′ss indicate that the engineered U1snRNAs are correctly expressed and assembled into functional U1snRNP module.

The majority (>99%) of introns are spliced out by the U2 spliceosome, with the canonical and highly conserved GT 5′ss (consensus sequence MAG/GURAGU, where M indicates A or C, and R, A or G) recognized by the 5′ tail of the U1snRNA. However, ~1% of U2-type introns have the GC dinucleotide at position +1/+2 of 5′ss and it is recognized that a higher complementarity with the U1snRNA is required to compensate the U to C substitution [26,27]. Moreover, while different studies demonstrated that changes within the canonical GT 5′ss can be efficiently rescued by engineered U1snRNAs [8,11,12,16,28], to our knowledge no attempt has been provided so far for nucleotide changes occurring within the GC 5′ss variant. Therefore, to investigate further the U1snRNA-mediated rescue at protein and activity level of splicing mutations in the peculiar GC context, we focused on *CDKL5* exon 3 c.99+5G>A mutation and developed a splicing-competent full-length *CDKL5* transgene. Moreover, since ExSpeU1s are predicted to possess higher exon specificity and reduced off-targets as compared to compensatory U1snRNAs [15], only the ExSpeU1s3 was considered. Notably, as shown by western blotting and functional analysis, the introduction of the c.99+5G>A mutation led to complete exon 3 skipping and to a shorter protein which is completely dysfunctional, in agreement with the crucial role of the CDKL5 ATP-binding domain encoded by exon 2 through 11. Since it has been previously proposed that shorter CDKL5 isoform might be characterized by defective subcellular distribution [29,30], we verified whether the exon 3-skipped CDKL5 isoform manifested any localization defect. However, as observed by the quantification of nuclear or cytoplasmatic CDKL5 signal, we did not observe any significant variation related to CDKL5 distribution. Noticeably, in this system, the co-transfection of the U1s3 was responsible for a significant and dose-dependent increase of full-length CDKL5 protein, whose kinase functionality was demonstrated by the pTEY assay. However, because of the neurological origin of the disease, by lentiviral transduction, we delivered into *Cdkl5* null hippocampal neurons the U1s3-coding cassette as well as the full-length *CDKL5* splicing-competent transgene. Rescue of *CDKL5* was evident both at mRNA level and in the amelioration of dendritic morphology, evaluated by Sholl analysis. Considering that the recovery of *Cdkl5* defective neuronal morphology is often considered a valid measurable outcome for the identification of novel therapeutic strategies, we find these results highly valuable.

Overall, our in vitro and ex vivo data elucidate the molecular mechanisms underlying the defective *CDKL5* expression in the presence of different nucleotide changes impairing the splicing process. Moreover, these findings provide the rationale for an approach based on U1snRNA variants able to rescue the splicing defects at RNA and protein levels, and able to revert typical *Cdkl5* null neuronal phenotypes. These results encourage further investigations in mouse models, generated to mimic *CDKL5* splicing defects, to properly evaluate the therapeutic potential of transducing viral particles expressing an ExSpeU1 cassette. To our knowledge, these findings provide the first proof-of-concept of the U1snRNA-mediated rescue of *CDKL5* splicing mutations and of nucleotide variants occurring within the non-canonical GC 5′ss.

## 4. Materials and Methods

### 4.1. Antibodies

The following antibodies were used for western blot and immunofluorescence experiments: mouse monoclonal anti-CDKL5 (for western blot) (sc-376314; Santa-Cruz Biotechnologies, Dallas, TX, USA), rabbit polyclonal anti-CDKL5 (for immunofluorescence) (HPA002847; Sigma-Aldrich, St. Louis, MO, USA), rabbit polyclonal anti-active Mitogen-Activated Protein Kinase (MAPK; used to detect the phosphorylated TEY motif; V8031, Promega, Madison, WI, USA), mouse monoclonal anti-α-tubulin (T6074; Sigma-Aldrich), mouse monoclonal anti-GFP (1814460; Roche Diagnostics, Basel, Switzerland), mouse monoclonal anti-GAPDH (G3171; Sigma-Aldrich).

### 4.2. Cell Cultures

HEK293T and HeLa cells were maintained in DMEM (Dulbecco’s Modified Eagle Medium) (Sigma-Aldrich) supplemented with 10% FBS (Euroclone, Milan, Italy), 1% l-glutamine (Sigma-Aldrich) and 1% penicillin/streptomycin (Sigma-Aldrich) at 37 °C with 5% CO_2_ in T75 flasks. When confluent, cells were washed with sterile 1× PBS (Sigma-Aldrich and passaged with 1× trypsin solution (Sigma-Aldrich), which was then inactivated with growth medium. Cells were centrifuged at 1200× *g* for 5 min and the cell pellet was disaggregated by pipetting in 1 mL of growth medium. Cells were seeded in 12-multiwell plates (Corning, Tewksbury, MA, USA) for RT-PCR and western blot or in 24-multiwell plates for immunofluorescence.

WT and c.2376+5G>A mutated Lymphoblastoid Cell Lines (LCLs) were maintained in RPMI medium-1640 (Roswell Park Memorial Institute) (Sigma-Aldrich), supplemented with 15% FBS, 1% L-glutamine, 1% penicillin/streptomycin, 2.5% HEPES (Euroclone) and 0.1% nystatin (Sigma-Aldrich) at 37 °C with 5% CO_2_ in 24-multiwell plates. Cells were seeded in 24-multiwell plates (Corning) for RT-PCR and western blot analyses (300,000 cells/well).

### 4.3. Primary Hippocampal Neurons

WT and *Cdkl5*^−/y^ primary hippocampal neurons were prepared from E18 CD1 mouse embryos isolated from heterozygous females. Mouse genotype was determined by PCR protocol on genomic DNA purified from ear punches using the following primers: forward primer for the null allele 5′-ACGATAGAAATAGACGATCAACCC-3′; forward primer for the WT allele 5′-CCCAAGTATACCCCTTTCCA-3′; common reverse primer 5′-CTGTGACTAGGGGCTAGAGA-3′. Dissected tissues were washed in HBSS (Sigma-Aldrich) and then incubated at 37 °C for 15 min in HBSS containing 0.1% trypsin (Life-Technologies, Carlsbad, CA, USA) and 0.05% DNase (Roche Diagnostics). The digestion was blocked with 10% FBS in DMEM and hippocampi were mechanically dissociated in 1 mL of dissecting medium with a sterile Pasteur pipette. Vital cells were counted using trypan blue and plated in 24-multiwell plates coated with poly-D-lysine (0.1 mg/mL) for RT-PCR or on poly-D-lysine coated coverslips (Neuovitro Corporation, Vancouver, WA, USA) for immunofluorescence at a density of 70,000 neurons/well in 10% FBS in DMEM. After 2 h at 37 °C to allow cell attachment, the medium was replaced with Neurobasal (Life-Technologies), supplemented with 2% B27 (Life-Technologies), 1% glutamine and 0.5% penicillin/streptomycin.

### 4.4. LCL Treatment with Cycloheximide 

WT and 2376+5G>A mutated LCLs were plated in 24-multiwell plates at a concentration of 70,000 cells/well and treated with 300 mg/mL of Cycloheximide (CHX) (Sigma-Aldrich), an inhibitor of NMD. After 4 h, cells were lysed in 300 µL Purezol reagent (Bio-Rad, Hercules, CA, USA) for RNA purification. Then, 700 ng of isolated RNA were retrotranscribed using the iScript cDNA synthesis kit (Bio-Rad). Splicing pattern was analysed by RT-PCR using the following primers: a common forward primer, 5′-CAACAGCCTGCAACTCTTGT-3′; a reverse primer (exon 15–18), 5′-TCGGAATTGGGTACCATGGA-3′; a reverse primer (exon 16–18), 5′-GCTTTGGGTCTGCAGATCTG-3′. rRNA 18S was determined by the following primers: forward primer, 5′-GTAACCCGTTGAACCCCATT-3′ and reverse primer, 5′-CCATCCAATCGGTAGTAGCG-3′.

### 4.5. Generation of Minigenes and Splicing Competent cDNAs 

For the minigene splicing assay, human *CDKL5* (h*CDKL5*) exons 3, 4, 7, 9 or 16 and approximately 500 bp of each flanking intronic sequence were cloned into the pTB vector (pTBNde (min) was a gift from Prof. Franco Pagani, Addgene plasmid #15125) using the unique site *NdeI*. Splicing mutations were inserted using the Q5 Site Directed Mutagenesis kit (New England Biolabs, Ipswich, MA, USA).

The WT or the c.99+5G>A h*CDKL5* splicing competent cDNAs (pSC-CDKL5WT-pSC-CDKL599+5A) were generated by sequential cloning in the pGEM-T easy vector (Promega). The first fragment was generated by PCR on genomic DNA (gPCR) and it is composed of h*CDKL5* Kozak sequence, followed by the ATG signal, exon 2 sequence and the initial part of intron 3, which bears the 5′ splicing donor site (5′ss). The amplicone was generated by using the following primers: forward, 5′-TGTATGCATCTCGAGAGTTTGTCTTCATGAAGATTCCT-3′ and reverse, 5′-TGTGAGCTCGAGACTCTGTCTTGAATGAATG-3′. PCR product was digested with NsiI/SacI and cloned in the pGEM-T easy vector. The second fragment was generated by PCR on the WT or c.99+5G>A mutated pTB exon 3 vector, by using the following primers: forward, 5′-TGTGAGCTCCGATCTGGACTACTGCAACC-3′ and reverse, 5′-TGTCATATGGTCATTATGAGGTGCACGGC-3′. This amplicone is composed of the terminal part of intron 3, comprising the 3′ splicing acceptor site (3′ss) and the branch point site (BS), followed by exon 3 and the initial part of intron 4, which carries the 5′ss. The PCR product was then digested with SacI/NdeI and cloned at the 3′ of the first fragment in the pGEM-T easy vector.

The third fragment comprises the terminal part of intron 4, bearing the 3′ss and BS, which was amplified by gPCR using the following primers: forward, 5′-TGTCATATGTTATCTCACTTTGGATTTGCAGTGG-3′ and reverse, 5′-ATCGCCACAATTTCATGTGTTTCC-3′. The sequence from exon 4 to exon 19 was amplified by PCR on the pEGFP-C1 h*CDKL5* vector [31] exploiting the following primers: forward, 5′-GGAAACACATGAAATTGTGGCGAT-3′ and reverse, 5′-TGTGCGGCCGCCGGATCCTTACAAGGCT-3′ and fused at the 3′ of intron 4 by overlap PCR. The resulting amplicone was digested using NdeI/NotI and inserted at the 3′ of the second fragment in the pGEM-T easy vector. Finally, the pSC-CDKL5WT and the pSC-CDKL599+5A were cloned in the pCAGIG vector by a double enzymatic digestion with XhoI/NotI.

### 4.6. Splicing Pattern Analysis

HEK293T cells were transfected using Lipofectamine 3000 (Invitrogen, Carlsbad, CA, USA) with 600 ng of WT or mutated pTB minigenes, together with 200 ng of GFP expressing plasmid (pEGFP-C1) to test transfection efficiency. U1snRNAs and ExSpeU1 expressing plasmids were transfected with a 1× molar ratio. The splicing pattern was analysed by RT-PCR with the primers α-2,3 Globin forward, 5′-CAACTTCAAGCTCCTAAGCCACTGC-3′ and Bra2 reverse, 5′-TAGGATCCGGTCACCAGGAAGTTGGTTAAATCA-3′.

To test the effect of +5 splicing mutation, HEK293T cells were transfected using Lipofectamine 3000 with 600 ng of pSC-CDKL5WT or pSC-CDKL599+5A, together with a 1× or 2.5× molar ratio of U1snRNAs and ExSpeU1. The splicing pattern was analysed by RT-PCR with the primers forward, 5′-TGTATGCATCTCGAGAGTTTGTCTTCATGAAGATTCCT-3′ and reverse, 5′-CCGATACCATCTGGTGGCACG-3′.

### 4.7. Lentiviral Production and Neuronal Infection

To create the lentiviral plasmid expressing the U1^s3^, the coding sequence of the U1^s3^ was cloned into a second-generation lentiviral plasmid backbone (pLV-PGK-EGFP, Cyagen Biosciences, Santa Clara, CA, USA) using the *BamHI-SalI* restriction sites. On the other hand, the lentiviral plasmid expressing the full-length splicing competent *CDKL5* transgene was generated by insertion of the *CDKL5* transgene into the pLentilox backbone (Addgene repository, plasmid #11795) through the *XhoI*-*NotI* restriction sites. To generate the lentiviral stock used to transduce hippocampal neurons, the pLV-U1^s3^ or pLenti-CDKL5 plasmids were co-transfected with the psPAX2 packaging and pMD2.G envelope plasmids into HEK293T cells. Virus supernatants were collected and lentiviral titer (2.67^+05^ vg/μL) was determined by fluorescence titering assay. Briefly, serial dilutions of lentiviral stock were used to infect 75,000 seeded cells. After 48 h post infection, cells were harvested, and the percentage of GFP-positive cells was evaluated by fluorescence-activated cell sorting (FACS) analysis.

Hippocampal neurons were infected at DIV1 with the lentivirus pLL SC-CDKL5WT, SC-CDKL599+5A alone or in combination with pLL ExSpeU1 and analyses conducted at DIV7. WT neurons were used for RT-PCR analysis, whereas *Cdkl5* null neurons were infected for immunofluorescence.

### 4.8. Western Blotting Analysis

Confluent HEK293T cells and LCLs were lysed in 120 µL of Laemmli Buffer. Following this, 20 µL of cell lysate were heated at 70 °C for 10 min and separated on a 10% SDS-PAGE. Proteins were blotted on a nitrocellulose membrane using a semidry transfer apparatus (Trans-blot SD; Bio-Rad) and membranes incubated 1 h in blocking solution (Tris-buffered saline containing 0.1% Tween-20 (TBS-T) and 5% non-fat dry milk). Membranes were incubated with primary antibody at the proper dilution: anti-phospho-TEY (1:2000 in 0.4% BSA in TBS-T), anti-α-tubulin (1:10,000 in 5% milk in TBS-T), anti-GFP (1:1000 in 5% milk in TBS-T) and anti-GAPDH (1:10,000 in 5% milk in TBS-T). After 3 washes in TBS-T, blots were incubated with the appropriate HRP-conjugated secondary antibody (1:10,000 in 5% milk in TBS-T) and the immunocomplexes visualized by using the ECL substrate kit (GeneSpin) and the Uvitec system (Cleaver Scientific, Warwickshire, UK). Band density measurements were performed using the Uvitec software. To detect total CDKL5, phospho-TEY signal was stripped by incubating the membrane in the stripping solution (StripAblot; Euroclone) for 15 min at RT and washed in TBS-T for 20 min. The membrane was incubated in blocking solution for 1 h and then with the anti-CDKL5 primary antibody (1:1000 in 5% milk in TBS-T). The phosphorylation status of CDKL5 was calculated as a ratio between the phospho-TEY and the total isoform of CDKL5. CDKL5 levels were calculated by using, as internal control, GFP in HEK293T or GAPDH in LCLs. α-tubulin served as loading control.

### 4.9. Immunofluorescence 

HeLa cells were washed 3 times with 1X PBS for 5–10 min and then fixed with 4% ice-cold paraformaldehyde (PFA, diluted in 1× PBS) for 15 min. After 3 washes in 1× PBS, cells were incubated for 5 h at RT in blocking solution (0.2% Triton-X100, 5% Horse Serum in 1× PBS). Cells were then incubated with anti-CDKL5 primary antibody (1:1000 in blocking solution) overnight at 4 °C and after 3 washes in 1× PBS, cells were incubated with the Alexa-fluor 568 donkey anti-rabbit secondary antibody (Life-Technologies) for 1 h at RT. After 3 washes in 1× PBS, DNA was counterstained with DAPI (1:1000 in 1× PBS; Life-Technologies) for 5 min and coverslips were mounted with Fluoromount-G reagent (Life-Technologies). Images were acquired from at least 3 coverslips for each experimental group under a confocal fluorescence microscope (Nikon, Tokyo, Japan) using an excitation wavelength of 568 nm with a 100× objective. Images were processed by Fiji software (www.fiji.sc) in order to calculate total, nuclear and cytoplasmic fluorescence.

Hippocampal neurons were fixed with ice-cold 4% PFA for 15 min and then washed 3 times in 1× PBS before incubation in 0.2% TritonX-100 in 1× PBS for 3 min on ice. After 3 washes with 0.2% BSA in 1× PBS at RT, cells were incubated in 4% BSA in 1× PBS for 15 min and then with the anti-GFP primary antibody overnight at 4°C (1:1000 in 0.2% BSA in 1× PBS). Cells were then incubated for 1 h with the Alexa-fluor 488 anti-mouse secondary antibody (Life-Technologies) and, after 3 washes in 1× PBS, DNA was stained with DAPI (1:1000 in 1× PBS) for 5 min. Cells were washed 8–10 times with 1× PBS and coverslips were mounted with Fluoromount-G reagent. GFP-positive neurons were acquired under an epi-fluorescence microscope (Nikon), equipped with a 40× objective and neuronal arborisation was analysed using a Sholl Analysis plug-in distributed with Fiji software.

### 4.10. Statistical Analysis

Data are expressed as mean ± SEM and were analyzed using Graph Prism software 7.0. Data of CDKL5 expression and subcellular localization were analyzed by one-way ANOVA, whereas morphological results from Sholl analysis in neurons were analyzed by two-way ANOVA and multiple comparison was performed by Tukey’s post-hoc test. Repeated measures one-way AVOVA was used for the statistical analysis of cellular growth on LCLs cells and multiple comparison was performed by Sidak’s post-hoc test. A *p* value < 0.05 was considered significant.
